# The value of combined thromboelastography test for the early prediction of disease severity in patients with haemorrhagic fever of renal syndrome and the construction of a critical model

**DOI:** 10.1371/journal.pone.0331930

**Published:** 2025-09-26

**Authors:** Longjiao Dou, Yanxia Huang, Haizhou Miao, Songchang Xiu, Fengmin Hu, Hongyi Chen, Daya Luo, Shumei Wang

**Affiliations:** 1 The First Department of Infectious Disease, The Affiliated Infection Hospital of Nanchang University, Nanchang, Jiangxi, China; 2 School of Clinical Medicine, Nanchang University, Nanchang University, Nanchang, Jiangxi, China; 3 The Department of Biochemistry and Molecular Biology, School of Basic Medical Sciences, Nanchang University, Nanchang, Jiangxi, China; Christian Medical College Vellore, INDIA

## Abstract

**Background:**

Thromboelostograms (TEG) are indicators that reflect the dynamic changes in blood coagulation objectively. Compared with traditional coagulation tests, TEG are easy to perform; imparting a head start in determining patients’coagulation status. The aim of this study was to explore the role of thromboelastography as an early predictor of disease severity and as a prognostic factor in patients with haemorrhagic fever of renal syndrome (HFRS).

**Methods:**

This was a retrospective study in which we collected clinical data from 342 patients with HFRS who were hospitalized from January 2017 to January 2021. The predictive value of laboratory parameters for HFRS criticalization was assessed via receiver operating characteristic (ROC) curves. After that, a model of criticalization was developed using stepwise analysis, subsequently, the model was evaluated and validated internally as well as externally. This study was approved by the Ethics Committee of the Hospital.

**Results:**

The study population was categorized into critical and noncritical groups according to their condition during hospitalization, with a median age of 52.00 (39.00–61.50) years for the critical group and 39.00 (16.00–53.25) years for the noncritical group. Both groups had a large proportion of male patients (65.3% and 72.3%, respectively). The median duration of hospitalization was 15.00 (3.50–25.00) days in the critical group and 13.00 (10.00–17.25) days in noncritical group. In the critical group 40.8% of patients died. The incidence of bleeding was greater in critical patients (51.0%) than in noncritical patients (15.1%). The coagulation indices: CI, K, MA, R and angle were significantly different between the two groups (p < 0.05). Critical patients had higher levels of K and R and lower levels of CI, MA and angle. Multivariate analysis revealed that K, ALB, CK, SCR, angle, WBC and LYM were independently associated with disease severity in patients with HFRS. ROC curve analysis revealed that the criticality model equation had an AUC of 0.9058, a sensitivity of 88.9% and a specificity of 80.2%, which were better than that of any single parameter in predicting the patient’s outcome. The internal validation C index was 0.866, the GOF P value was 0.825, and the external validation AUC was 0.762, with a sensitivity of 71.4% and a specificity of 74.2%. The results of the calibration plots indicated that there was some agreement between the model-estimated HFRS critical illness rates and the final observed critical illness rates in the study population. Decision curve analysis indicated that the model had significant clinical utility and comparable net benefit over a range of threshold probabilities.

**Conclusion:**

Critical HFRS have significantly abnormal coagulation function and high incidence of bleeding associated with poor outcome. A criticalization prediction model constructed on the basis of thromboelastography indices in early stages of the disease has a good predictive ability and may be helpful in the early identification of critically ill patients for timely clinical intervention and treatment.

## Introduction

Haemorrhagic fever of renal syndrome (HFRS) is an acute zoonotic infectious disease spread by rodents and caused by Hantaan virus (HTNV), Seoul virus (SEOV), Puumala hantavirus (PUUV) and other serotypes of Hantavirus [1,2]. It is estimated that more than one million cases of HFRS occur worldwide each year, but only approximately a quarter of the actual infections are diagnosed and reported because of population mobility, difficulties in tracking infections, and the fact that many asymptomatic or mildly ill infections are undiagnosed and unreported [3–5]. In recent decades, China has been burdened with the most severe outbreaks of HFRS, accounting for 70% to 90% of the global incidence of the disease [2]. A mean of 12 800 HFRS cases (median 11 063; range 8853–25 041) per year was reported in China from 2004 to 2016, with a case fatality rate (CFR) of 1·3% [6]. HTNV and SEOV are the main causes of HFRS in China, and patients infected with HTNV experience more severe disease, with a mortality rate of 5–10% [7,8]. The clinical manifestations of patients infected with hantaviruses vary from subclinical, mild, and moderate to severe, and those with severe disease urgently require intensive care treatment. However, easily actionable biomarkers or clinical parameters for the early risk stratification of HFRS patients are still lacking. With the increasing implementation of preventive measures, including vaccinations, and improvements in effective and supportive treatments over the past few decades, the incidence of HFRS has declined in China in recent years [6],. However, HFRS is still an important infectious disease with relatively high morbidity and mortality in China. Therefore, clinical parameters that can be used to evaluate the severity and predict the disease course of HFRS are of great significance for improving the management of patients with HFRS.

Bleeding symptoms are among the main clinical features of HFRS. Some studies have reported mild bleeding in one-third of patients with PUUV, and in HFRS caused by HTNV and Dobrava virus (DOBV), haemorrhage is more common in severe cases [9] Haemorrhagic manifestations of HFRS, which range from petechiae to severe internal haemorrhage, may include skin and mucous petechiae, ecchymosis, conjunctival congestion, vomiting blood, epistaxis, haematuria, black stools, and fatal intracranial haemorrhage [10,11].Hantavirus infection induces haematological abnormalities such as destruction of vascular endothelial cells, accelerate platelet activation, and increased fibrinolysis and complement activation [12–15], which ultimately disrupts the balance between haemorrhage and haemostasis, leading to haemorrhage, thrombosis and even disseminated intravascular coagulation (DIC), and ultimately to multiorgan dysfunction or failure. Therefore, both the pathophysiological and clinical manifestations that occur during hantavirus infection indicate that the changes in coagulation status may be related to the clinical development of the disease. Theoretically, thromboelastography (TEG) can realize real-time continuous monitoring of coagulation status by rapidly detecting dynamic changes in blood coagulation, such as coagulation factor activation, platelet aggregation, and fibrin formation. Compared with traditional coagulation test (e.g., prothrombin time (PT) and activated partial thromboplastin time (APTT), TEG does not require the processing of blood samples, is easy to perform, can be used on whole blood or plasma without the need for processing in a standard laboratory; and can quickly obtain results at the bedside; can integrate plasma and cellular impact on coagulation; and provides a more realistic and objective evaluation of the entire coagulation process, from the initiation of coagulation through, thrombus formation, to thrombolysis. Existing tests (e.g., PT, INR and APTT), which require the processing of blood samples and monitor only one point or part of the coagulation or fibrinolytic process, are less useful in monitoring patients with haemorrhagic fever, so TEG may be a better reference for managing these patients. Currently, TEG is mostly used to guide clinical blood transfusion, coagulation monitoring during surgery and adjustment of anticoagulation and antiplatelet therapy. With respect to HFRS, the effect and value of TEG application have rarely been reported. Therefore, the aim of this study was to analyse the clinical significance of TEG index levels within 24 hours of admission in HFRS patients, as well as to construct a risk-stratified prediction model with the ability to identify HFRS criticality.

## Methods

### Patients

We reviewed the medical records of 342 patients with HFRS who were continuously hospitalized at the Infection Hospital affiliated with Nanchang University from January 2017 to January 2021, and the data included demographic characteristics, clinical characteristics, and laboratory data within 24 hours of admission. All enrolled patients met the following inclusion criteria: meeting the diagnostic criteria in China’s *Expert Consensus on Prevention and Treatment of haemorrhagic fever with renal syndrome (2021 edition)* (patients who met the clinical diagnosis or who had been diagnosed with a confirmed diagnosis (positive serology-specific IgM antibody or detected hantavirus RNA or a more than 4-fold increase in serum specific IgG antibody titer in the recovery phase compared with that in the acute phase)).Patients were excluded according to any of the following criteria: (1) complicated with severe cardiovascular and cerebrovascular diseases; (2) congenital coagulation system diseases and platelet genetic related diseases; (3) long-term use of anticoagulant and antiplatelet drugs; (4) history of venous thromboembolism; (5) incomplete or incorrect clinical information; and (6) other diseases affecting coagulation function, such as pregnancy, chronic kidney disease, autoimmune disease, chronic viral hepatitis, cirrhosis, COPD, silicosis, tuberculosis or other infectious diseases. The medical records of 259 HFRS patients from January 2017 to January 2020 were used for construction of the model, and the medical records of 83 HFRS patients from January 2020 to January 2021 were used for external validation.

Informed consent from patients was not obtained because patient records and information had been anonymized and deidentified prior to the study. After review by the Ethics Committee of the Affiliated Infection Hospital of Nanchang University, the informed consent process has been exempted. This study was approved by the Ethics Committee of Infection Hospital Affiliated to Nanchang University (reference number 202319). Case data acquisition began on December 1, 2023.

### Grouping and definition

According to China’s “*Expert Consensus on prevention and treatment of haemorrhagic fever with renal syndrome (2021 edition)*”, HFRS is classified according to the severity of illness during hospitalization. The specific clinical classification criteria are shown in **Table 1**. To analyse factors associated with disease severity, patients were divided into two groups based on the classification (noncritical group: mild, moderate, and severe patients; critical group: critical patients). Serological diagnosis of HFRS was confirmed by positive results of HTNV-specific IgM/IgG capture ELISA.

**Table 1:** Grouping criteria based on the HFRS^a^ clinical classification criteria.

classification	Conditions
Mild	body temperature was below 39 °C,there were skin mucosal haemorrhagic spots, urine protein was “+ to ++”, no oliguria and hypotensive shock.
Moderate	The body temperature was 39–40 °C, the bulbar conjunctival edema was obvious, the skin and mucosa had obvious ecchymosis, the systolic blood pressure was lower than 90 mmHg(1 mmHg = 0.133 kPa) or the pulse pressure difference was less than 30 mmHg during the course of the disease, oliguria, and the urine protein was “++ to +++”.
Severe	Body temperature above 40 °C, neurological symptoms, shock, oliguria for 5 days or anuria for less than 2 days.
Critical	On a severe basis, one of the following conditions, refractory shock, vital organ bleeding, no urine for more than 2 days, other serious comorbidities such as heart failure, pulmonary edema, respiratory failure, coma, secondary serious infection.

a: The clinical classification criteria for HFRS are derived from China’s *Expert Consensus on Prevention and Treatment of haemorrhagic fever with renal syndrome (2021 edition)*.

### Data collection

In this study, blood samples were collected from 342 patients with HFRS within 24 hours of admission. Routine laboratory tests (routine blood tests, biochemical tests and blood clotting tests) were performed in the clinical laboratory as instructed by the attending physician. Age, gender,

days from fever to hospital admission and hospital days were extracted from electronic medical records. Clinical signs and symptoms include haemoptysis, haematemesis, black stool, haematuria, ecchymosis, haematoma, and rupture or bleeding of internal organs. Other concomitant diseases included hypertension, diabetes, chronic hepatitis B, cirrhosis, chronic kidney disease, uraemia, Sjogren’s syndrome, tuberculosis, etc. Routine laboratory parameter test results included white blood cell count (WBC), red blood cell count (RBC), haemoglobin (Hb), haematocrit (HCT), platelet (PLT), neutrophil count (NE), lymphocyte count (LYM), procalcitonin (PCT), C-reactive protein (CRP), alanine aminotransferase (ALT), aspartate aminotransferase (AST), total bilirubin (TBil), albumin (ALB), triglyceride (TG), lactate dehydrogenase (LDH), creatine kinase (CK), creatine kinase isoenzyme (CK-MB), blood urea nitrogen (BUN), and serum creatinine (Scr). TEG mainly included the composite clotting index (CI), reaction time (R) value, K value, α angle, maximum amplitude (MA) value, fibrinolytic index (LY30), and fibrinolytic index (EPL). The R value is equivalent to the thromboplastin generation time, which is the time needed to detect the initial clot formation in the blood sample. The R value is prolonged due to the lack of coagulation factors, decreased coagulation factor activity or the presence of anticoagulants and is shortened in the hypercoagulable state. The K value is equivalent to the time of thrombin generation and indicates the speed of clot formation. The α angle is a parameter reflecting the rate of clot formation, which is mainly affected by the quantity or quality of fibrinogen thrombin and platelets. Both the K value and α angle reflect the rate of blood clot formation. When the R value or K value is higher or the α angle or MA value is lower than normal, the patient is considered to have a hypocoagulable state.

### Statistical analysis

The Kolmogorov‒Smirnov test was used to test the normality of measurement data. For comparison between the two groups, the normally distributed data were expressed as Mean ± standard deviation (M ± SD) by the t test. The nonnormally distributed data were measured by the Mann‒Whitney test and expressed as interquartile spacing(M(P2 ~ P7)). Count data are expressed as percentages and were tested by the chi-square test (χ2) or Fisher’s Precision Test for analysis. The predictive value of the laboratory parameters was tested using receiver operating characteristic (ROC) curves and quantified by calculating the area under the ROC curve (AUC) and 95% confidence interval (CI). The multicollinearity test assessed the linear correlation between the independent variables. Logistic regression analysis was used for the analysis of risk factors associated with HFRS criticalization, and stepwise approach analysis was used for model development. The final model differentiated and quantified the results using calibration plots, Goodness of fit (GOF) and C-index estimates. External validation was temporal validation and quantification was performed by calculating the area under the ROC curve (AUC) and 95% confidence intervals (CI). p < 0.05 was considered a statistically significant difference.

## Results

### Demographic information of the study population

From January 2017 to January 2020, 281 patients with HFRS were admitted to the treatment centre. After applying the inclusion criteria, 22 patients were excluded, and a total of 259 patients were included in this study. Among them, 184 (71.04%) were male, and 75 (28.95%) were female. There were 20 deaths, 40.8% of critical patients. According to the severity grouping criteria, 72 cases were mild, 101 were moderate, 37 were severe, meaning the total number of patients in the noncritical group was 210, and the total number in the critical group was 49. The mean age of patients in the critical group was 52.00 (39.00–61.50) years, significantly (P < 0.05) older than that of patients in the noncritical group (mean age 39.00 (16.00–53.25) years). The mean duration of fever to hospital admission was 5.00 (4.00 ~ 7.00) days in the critical group and 5.00 (4.00 ~ 7.00) days in the noncritical group, and there was no statistically significant difference between the two groups in terms of sex and days from fever to hospital admission (P > 0.05). The average number of days of hospitalization was 15.00 (3.50 ~ 25.00) days in the critical group, and 13.00 (10.00 ~ 17.25) days in the noncritical group, and the difference between the two groups was not statistically significant (P > 0.05) because 20 patients in the critical group died shortly after admission. Among the symptoms of dread cold and fever, headache, body ache, fatigue, nausea and vomiting, lumbar pain, abdominal pain, bloating, and haemorrhagic manifestations (Petechiae, ecchymosis, haematuria, black stools, haemoptysis, hematoma), only the incidence of haemorrhage in the critical group (51.0%) was significantly greater than the incidence of haemorrhage in the noncritical group (15.1%). (Table 2)

**Table 2:** Clinical features of HFRS patients in the training cohort.

Parameters	noncritical (n = 210)	critical (n = 49)	χ^2^/Z	P
Male, n(%)	210/152	49/32	0.967	0.326
Age, years	39.00(16.00 ~ 53.25)	52.00(39.00 ~ 61.50)	−4.010	0.001
Days from fever to hospital admission, days	5.00(4.00 ~ 7.00)	5.00(4.00 ~ 7.00)	−0.080	0.937
Hospital days, days	13.00(10.00 ~ 17.25)	15.00(3.50 ~ 25.00)	−0.241	0.810
dread cold and fever	209(99.5%)	48(97.9%)	1.269	0.348
have a headache	82(39.0%)	29(59.1%)	6.578	0.010
body ache	135(73.8%)	34(69.3%)	0.456	0.499
fatigue	55(26.1%)	10(20.4%)	0.707	0.401
nausea and vomiting	85(40.4%)	5(10.2%)	16.057	0.001
lumbar pain	97(46.1%)	6(12.2%)	19.113	0.001
abdominal pain	41(19.5%)	6(12.2%)	1.417	0.234
bloating	53(25.2%)	13(26.5%)	0.035	0.852
Haemorrhage (Petechiae, ecchymosis, haematuria, black stools, haemoptysis, hematoma)	33(15.1%)	25(51.0%)	28.496	0.001

### Correlations between laboratory parameters and TEG indices of HFRS patients and disease severity

In noncritical group, the median value of WBC was 11.15(7.29 ~ 17.80)×109/L, the median value of PLT was 44.00(27.00 ~ 70.25)×109/L, the median value of NE was 6.06(3.98 ~ 10.51)×109/L, the median value of LYM was 2.55(1.47 ~ 4.98)×109/L, the median value of LDH median value was 691.00(497.25 ~ 918.50)U/L, CK median value was 123.00(61.75 ~ 266.00)U/L, CKMB median value was 30.10(19.45 ~ 46.42)U/L, BUN median value was 9.84(5.66 ~ 18.68) mmol/L, Scr median value was 124.00(80.50 ~ 296.00)umol/L, median value of AST was 114.70(71.67 ~ 190.90)U/L, median value of ALT was 68.55(42.07 ~ 124.50)U/L, median value of ALB was 29.91 ± 4.23g/L and median value of PCT was 1.73(0.89 ~ 4.67)ng/ml. In critical group, the median values of WBC were 22.85(18.15–37.64)×109/L, PLT were 29.00(18.00–37.5)×109/L, NE were 14.14(8.82–24.81)×109/L, LYM were 4.25(2.93–6.91)×109/L, LDH were 988.00(708.00 ~ 1490.00)U/L,CK were 351.00(159.00 ~ 733.50)U/L, CKMB were 49.20(34.95 ~ 83.55)U/L, BUN were 19.17(9.99 ~ 25.81)mmol/L, Scr were 329.00(144.00 ~ 516.00)umol/L, AST were 218.00(116.00 ~ 508.00)U/L, ALT were 85.00(61.00 ~ 208.00)U/L, ALB were 25.71 ± 5.56g/L, and PCT were 5.86(2.52 ~ 9.34)g/L. The critical group had significantly greater WBC, NE, LYM, LDH, CK, CKMB, BUN, Scr, AST, ALT and PCT, and lower PLT and ALB than did the control group (P < 0.05). The results of the above experimental indices revealed that the inflammatory response was more severe in critically ill patients than in noncritically ill patients. Possibly because of severe plasma leakage, haemorrhage and/or deep shock, and the tissues were in a state of hypoperfusion and hypoxic, which led to the multiorgan damage, such as cardiac, renal and hepatic injuries, that may be more severe in critically ill patients than in noncritically ill patients [16,17]. In non-critical group, the median value of CI was −2.10(−5.82 ~ 1.00), LY30 was 0.00(0.00 ~ 0.00) %, K was 3.10(1.90 ~ 5.42)min, EPL was 0.00(0.00 ~ 0.00)%, MA was 50.51 ± 13.41 min, R was 5.50(3.87 ~ 7.20)s and Angle was 55.90(42.02 ~ 65.75)°, respectively, for critical group, the median value of CI was −6.70(−12.70 ~ −1.40), LY30 median value was 0.00(0.00 ~ 0.00)%, K median value was 5.50(2.80 ~ 10.10)min, EPL median value was 0.00(0.00 ~ 0.00)%, MA median value was 42.00 ± 16.07 min, R median value was 6.90(4.90 ~ 9.80)s and Angle median value was 41.80(26.00 ~ 58.60)°. The differences between the two groups in the thromboelastographic indices CI, LY30, K, EPL, MA, R and angle were statistically significant (P < 0.05). In terms of the overall significance of the median level on the TEG index, patients in the critical group demonstrated a state of low platelet function and low fibrinogen function. Their noncritical group also showed a state of low fibrinogen function. The median PLT value in critically ill patients was 29.00(18.00–37.5)×109/L, which was lower than the median PLT value in noncritically ill patients, which was 44.00(27.00–70.25)×109/L. Therefore, critically ill patients were at greater risk of bleeding, and the bleeding might be more serious. There was no significant difference in RBC, HB, HCT, UA, TBIL, TG or CRP levels between the two groups (P > 0.05). (Table 3)

**Table 3:** Comparison of laboratory parameters of first medical contacts in a training cohort of HFRS patients.

Parameters	noncritical (n = 210)	critical (n = 49)	t/Z	P
WBC × 10^9^/L	11.15(7.29 ~ 17.80)	22.85(18.15 ~ 37.64)	−6.616	0.001
RBC × 10^9^/L	4.68 ± 0.77	4.80 ± 1.07	−0.712	0.479
HB g/L	136.65 ± 24.18	142.77 ± 31.83	−1.496	0.136
HCT%	39.62 ± 6.98	41.82 ± 9.16	−1.865	0.063
PLT × 10^9^/L	44.00(27.00 ~ 70.25)	29.00(18.00 ~ 37.5)	−4.234	0.001
NE × 10^9^/L	6.06(3.98 ~ 10.51)	14.14(8.82 ~ 24.81)	−6.127	0.001
LYM × 10^9^/L	2.55(1.47 ~ 4.98)	4.25(2.93 ~ 6.91)	−4.514	0.001
LDH U/L	691.00(497.25 ~ 918.50)	988.00(708.00 ~ 1490.00)	−4.511	0.001
CK U/L	123.00(61.75 ~ 266.00)	351.00(159.00 ~ 733.50)	−5.112	0.001
CKMB U/L	30.10(19.45 ~ 46.42)	49.20(34.95 ~ 83.55)	−5.035	0.001
BUN mmol/L	9.84(5.66 ~ 18.68)	19.17(9.99 ~ 25.81)	−4.596	0.001
Scr umol/L	124.00(80.50 ~ 296.00)	329.00(144.00 ~ 516.00)	−4.840	0.001
UA umol/L	348.00(239.00 ~ 490.00)	341.00(247.00 ~ 503.00)	−0.374	0.709
ALT^a^ U/L	68.55(42.07 ~ 124.50)	85.00(61.00 ~ 208.00)	−3.981	0.001
AST^a^ U/L	114.70(71.67 ~ 190.90)	218.00(116.00 ~ 508.00)	−2.473	0.013
TBIL^a^ umol/L	11.50(8.20 ~ 14.57)	13.10(7.90 ~ 16.50)	−1.204	0.229
ALB^a^ g/L	29.91 ± 4.23	25.71 ± 5.56	5.776	0.001
TG^b^ mmol/L	2.36(1.46 ~ 3.31)	2.59(1.71 ~ 3.56)	−0.553	0.580
PCT^c^ ng/ml	1.73(0.89 ~ 4.67)	5.86(2.52 ~ 9.34)	−4.222	0.001
CRP^d^ mg/L	24.25(12.98 ~ 36.69)	27.33(17.95 ~ 40.79)	−1.320	0.187
CI	−2.10(−5.82 ~ 1.00)	−6.70(−12.70 ~ −1.40)	−3.384	0.001
LY30%	0.00(0.00 ~ 0.00)	0.00(0.00 ~ 0.00)	−2.289	0.022
K min	3.10(1.90 ~ 5.42)	5.50(2.80 ~ 10.10)	−3.502	0.001
EPL%	0.00(0.00 ~ 0.00)	0.00(0.00 ~ 0.00)	−2.095	0.036
MA min	50.51 ± 13.41	42.00 ± 16.07	3.633	0.001
R s	5.50(3.87 ~ 7.20)	6.90(4.90 ~ 9.80)	−2.430	0.015
Angle°	55.90(42.02 ~ 65.75)	41.80(26.00 ~ 58.60)	−3.030	0.002

Note: a: 2 patients lacked data; b: 28 patients lacked data; c: 9 patients lacked data; d: 12 patients lacked data; thromboelastography data were missing for 21 patients.

### ROC curves for prediction of prognosis by single laboratory parameters

ROC curve analysis was used to explore the predictive value of laboratory parameters related to disease severity and TEG indices for prognosis (critical) in HFRS patients (**Table 4**). ALT, LY30 and EPL had no statistical significance in predicting prognosis (critical) (p > 0.05), while other indices had statistical significance (p < 0.05), the CI, K, MA, R, and angle° cutoffs were −5.45, 8.10 min, 47.35 min, 6.35 s, and 43.65°, respectively. However, almost all of the indicators with predictive value for critical illness had AUC values around 0.7.

**Table 4:** Predictive value of laboratory parameters for prognosis (critical severity) in HFRS patients.

Parameters	AUC	P- value	CUT-OFF value	Sensitivity	Specificity	95%CI FOR AUC	
						Lower	Upper
WBC × 10^9^/L	0.772	0.001	18.54	69.44%	79.01	0.704	0.854
PLT × 10^9^/L	0.691	0.001	35.5	75%	61.63%	0.216	0.401
NE × 10^9^/L	0.741	0.001	7.065	91.67%	56.98%	0.662	0.835
LYM × 10^9^/L	0.657	0.002	3.31	69.44%	63.37%	0.583	0.749
LDH U/L	0.703	0.001	815	69.44%	67.44%	0.607	0.798
CK U/L	0.668	0.001	293	61.11%	75.58%	0.592	0.803
CKMB U/L	0.694	0.001	33.7	77.78%	56.4%	0.602	0.785
BUN mmol/L	0.718	0.001	9.6	86.11%	49.42%	0.628	0.808
Scr umol/L	0.738	0.001	441.5	47.22%	88.95%	0.649	0.820
ALT U/L	0.604	0.051				0.497	0.710
AST U/L	0.664	0.002	129.3	72.22%	56.4%	0.559	0.769
ALB g/L	0.699	0.001	26.80	63.89%	77.91%	0.192	0.400
PCT ng/ml	0.710	0.001	5.17	61.11%	79.65%	0.614	0.806
CI	0.669	0.001	−5.45	58.33%	72.09%	0.223	0.438
LY30%	0.555	0.298				0.347	0.542
K min	0.673	0.001	8.10	44.44%	88.95%	0.567	0.786
EPL%	0.549	0.351				0.354	0.548
MA min	0.686	0.001	47.35	75.00%	56.40%	0.213	0.415
R s	0.624	0.019	6.35	61.11%	64.53%	0.517	0.732
Angle °	0.654	0.003	43.65	61.11%	69.77%	0.229	0.455

*Note: Moving to the next step, the candidates for use as prognostic predictors were those that differed by clinical type grouping.*

### Construction of risk models and validation of nomogram

**Fig 1:**
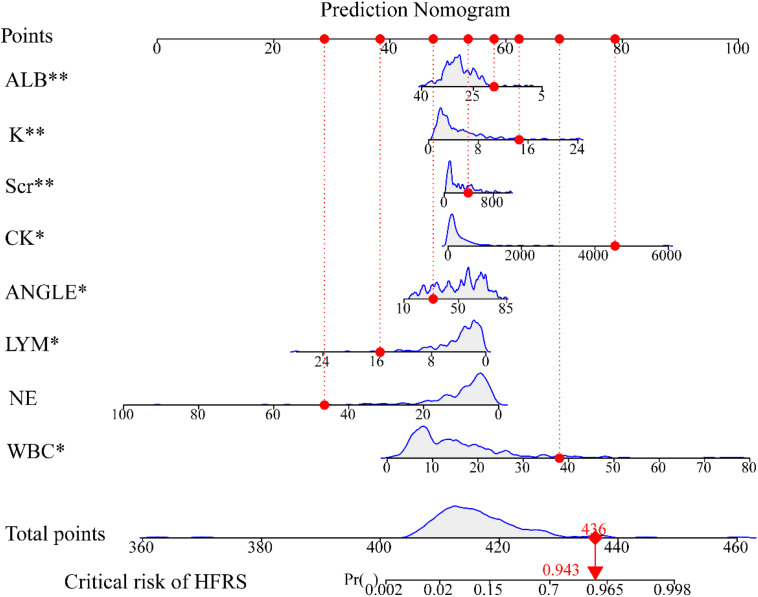
Nomogram of critical probability model in HFRS patients.

**Fig 2:**
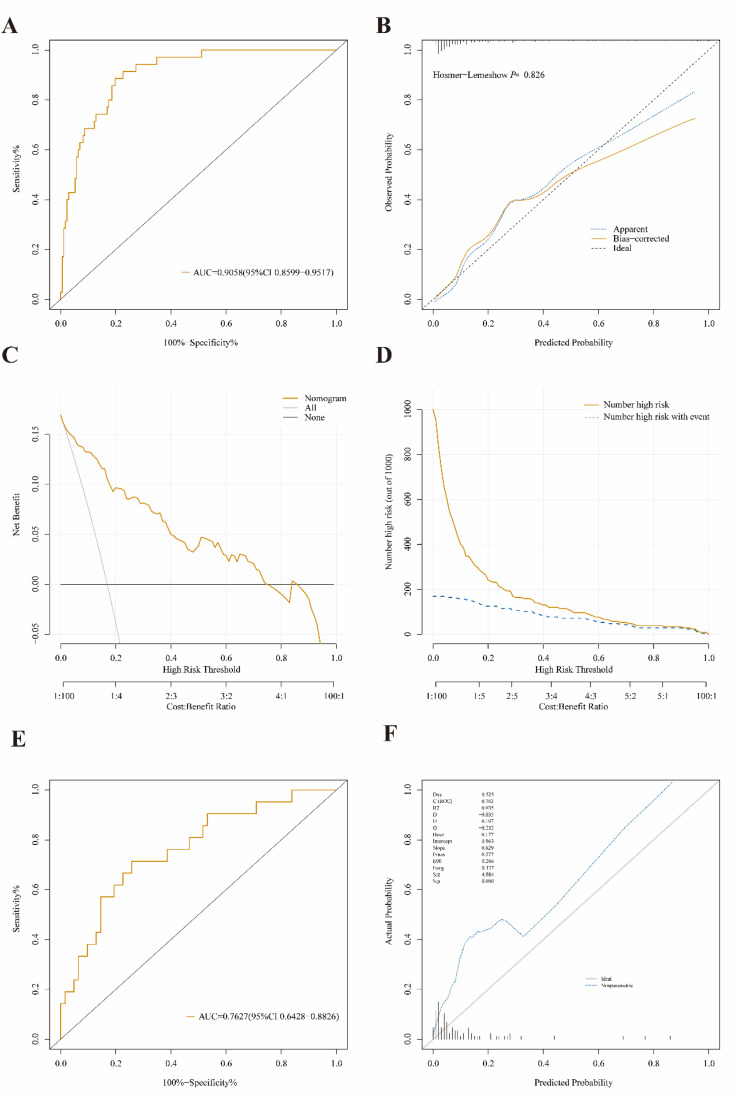
Internal and external validation of models.

Through multivariate logistic regression analysis, critical and noncritical stratification was defined as 1 and 0, respectively. The following parameters were independently associated with the development of criticalization: K (OR: 1.315; 95% CI: 1.094–1.630; P < 0.05), ALB (OR: 0.859; 95% CI: 0.779–0.937; P < 0.05), CK (OR: 1.001; 95% CI: 1.000–1.003; P < 0.05), SCR (OR: 1.002; 95%CI: 1.000–1.004; P < 0.05), Angle (OR: 1.062; 95%CI: 1.003–1.129; P < 0.05), WBC (OR: 1.221; 95%CI: 1.047–1.457; P < 0.05) and LYM (OR: 0.741; 95% CI: 0.540–0.971; P < 0.05) (Table 5). And the odds ratio (OR) of each parameter suggested that high levels of CK, Scr, WBC, K and Angle, and low levels of ALB and LYM, were risk factors for prognosis (critical) (**Fig 1**).After stepwise regression, a prediction model was constructed, this model has good value in identifying the critical condition of HFRS patients with initial medical contact, with an AUC of 0.9058 (**Fig 2A**), sensitivity 88.9%, specificity 80.2%. Based on internal validation used 1000 bootstrap resamples, internal validation showed good stability of the model with C-index = 0.866 and GOF P value = 0.825. The calibration plot visualization showed good agreement between the model-estimated incidence of critical condition and the observed incidence of critical condition in the study population (**Fig 2B**). The decision curves demonstrated that using a predictive model to predict the occurrence of critical HFRS situations and to inform intervention strategy adds more benefit than the ‘intervention-for-all’ scheme or the ‘intervention-for-none’ scheme when the risk thresholds range from 0% to 70% in the training cohort(**Fig 2C**). CIC curves, with risk thresholds >40%, showed that the model-predicted critical condition and the actual critical condition occurrences were highly matched, clinical prediction efficiency was high (**Fig 2D**). The model was externally validated using data from different periods (83 HFRS patients), and the validation result AUC was 0.762 (**Fig 2E**), sensitivity 71.4% and specificity 74.2%. The results of the calibration curves indicated that the external validation cohort fits the predictive model well. There was some agreement between the predicted incidence of critical status and the actual observed incidence of critical status in the validation cohort (**Fig 2F**).

**Table 5:** Logistic regression analysis of critical condition in patients with HFRS.

	Univariate analysis		Multivariate analysis	
Parameters	OR(95%CI)	P	OR(95%CI)	P
WBC × 10^9^/L	1.061(1.031-1.091)	0.001	1.221(1.047-1.457)	0.019
PLT × 10^9^/L	0.992(0.981-1.003)	0.144		
NE × 10^9^/L	1.047(1.016-1.079)	0.003	0.847(0.693-1.002)	0.088
LYM × 10^9^/L	1.069(0.981-1.165)	0.129	0.741(0.540-0.971)	0.044
ALT U/L	1.003(1.001-1.006)	0.007		
AST U/L	1.002(1.001-1.003)	0.001		
ALBg/L	0.864(0.799-0.993)	0.001	0.859(0.779-0.937)	0.001
PCTng/ml	1.022(0.997-1.048)	0.090		
LDH U/L	1.002(1.001-1.003)	0.001		
CK U/L	1.002(1.001-1.003)	0.001	1.001(1.000-1.003)	0.020
CKMB U/L	1.022(1.010-1.033)	0.001		
BUNmmol/L	1.083(1.044-1.123)	0.001		
SCRumol/L	1.003(1.002-1.005)	0.001	1.002(1.000-1.004)	0.004
LY30%	0.629(0.322-1.230)	0.176		
Kmin	1.160(1.076-1.251)	0.001	1.315(1.094-1.630)	0.006
EPL%	0.157(0.012-2.066)	0.159		
MAmin	0.950(0.923-0.979)	0.001		
Rs	1.129(1.027-1.240)	0.012		
Angle°	0.964(0.942-0.986)	0.001	1.062(1.003-1.129)	0.043

*Note: The CI value was calculated from the combination of R, K, α Angle and MA, so it was not included in the multivariate logistic regression analysis. ALT and AST VIF > 10, and ALT was included as an indicator in multifactorial analysis. Logit(P) =−3.866 + 0.199WBC-0.165NE-0.299LYM + 0.060Angle + 0.274K + 0.002 SCR + 0.001CK-0.154ALB.*

## Discussion

The results of our data analysis of the training cohort revealed that critically ill patients were, on average, older and had a higher incidence of bleeding than noncritical patients did, with a mortality rate of 40.8%. Differences in laboratory indices examined at the first medical contact between the two groups were statistically significant for WBC, NE, LYM, LDH, CK, CKMB, BUN, Scr, AST, ALT, PCT, PLT, ALB, CI, LY30, K, EPL, MA, R, and angle. Among them, except for ALT, LY30 and EPL, all the other indices had the moderate ability to predict the progression to critical illness (the AUC values were mostly approximately 0.7). On this basis, we further analysed the factors associated with the patients’ critical illness and found that K, ALB, CK, SCR, angle, WBC and LYM were independent risk factors for critical HFRS. As a result, a model equation for criticalization with good predictive value was constructed (AUC of 0.9058, sensitivity of 88.9%, and specificity of 80.2%). This model can help clinicians make initial judgments at the first medical contact on whether HFRS patients are at risk of progressing to critical illness.

The majority of the HFRS patients in this study were male patients, who were approximately 2.45 times more common than female patients. The overall mortality rate was 7.72%. In critically ill patients, the mortality rate was 40.8%. The average length of time from fever to hospital admission was consistent between critically ill patients and noncritically ill patients, with a mean of 5.00 (4.00–7.00) days, which coincides with the theoretical progression of the disease process to the oligohydramnios stages. This finding is consistent with what has been reported in previous studies [18]. Older individuals were more likely to have critical HFRS, bleeding was more common in severe cases, and the probability of an adverse outcome (death) was high in critically ill patients.

Our study confirmed that several laboratory markers, including WBC, NE, LYM, LDH, CK, CKMB, BUN, Scr, AST, ALT, PCT, PLT, and ALB, are associated with HFRS disease severity (P < 0.05). The leukocyte count has long been identified as a predictive marker for severe HFRS and a poor outcome (death) [19–21], whereas thrombocytopenia has been found to be associated with a more severe disease course [22,23]. Studies abound in which elevated markers representing renal impairment (e.g., Scr and BUN) have been correlated with HFRS disease severity and even prognosis [8]. The serum ferritin and PCT levels were significantly higher in critically ill patients than in mildly ill patients, which was correlated with HFRS severity, in a study by Lihe Che et al [24,25]. Liver injury is also common in patients with HFRS, and one study reported that the aspartate aminotransferase-to-platelet ratio index (APRI) can be used as a biomarker for identifying HFRS patients at risk for poor prognosis [26].In contrast, relatively few studies have investigated LDH, CK, and CKMB and the severity of HFRS.

Notably, all indices of coagulation, including CI, LY30, K, EPL, MA, R, angle assessed by thromboelastography, were significantly different between the critical and noncritical groups in our study (P < 0.05). The levels of K, R, CI, MA and angle in patients with HFRS varied with disease exacerbation, with K and R increasing and CI, MA and angle decreasing. In terms of the overall significance of the median levels of TEG indices, patients in the critical group exhibited a state of low platelet function and low fibrinogen function. The noncritical group exhibited a state of low fibrinogen function, and critically ill patients were more likely to have bleeding manifestations (Tables 3 and 4). Therefore, patients with HFRS not only have reduced platelet counts and fibrinogen depletion but also have diminished platelet function, fibrinogen function, and coagulation factor activity, leading to a state of hypocoagulability. Consistent with our conclusions, Koskela S.M. et al. revealed that thrombin activation and reduced thrombin production potential during the acute phase of HFRS via a calibrated automated thrombography (CAT). Combined with signs of low platelet count, enhanced fibrinolysis, and increased thrombin production in vivo, results in mild to moderate consumptive coagulation dysfunction, with a shift in the overall haemostatic balance towards a hypocoagulable state and bleeding tendency [27]. Wen-Jing Chen et al. reported that patients with fatal HFRS presented a longer PT, APTT, higher levels of D-dimer and fibrinogen degradation products (FDP), and lower levels of fibrinogen (Fib) [28]. During the acute phase of PUUV infection, there is an increase in the levels of circulating prothrombin fragment 1 + 2 (F1 + 2), fibrin degradation products and D-dimers, and there are decreases in the content of the physiological anticoagulants (AT), protein S (PS), and protein C (PC) and, suggesting increased thrombin formation and coagulation activation in vivo [1,9,29]. Following HTNV infection, TF expression is increased in endothelial cells, which can be mediated through the TF (i.e., exogenous pathway) and the contact plasma kinin-releasing enzyme–kinin system (i.e., endogenous pathway) induces coagulation and activates proinflammatory factors [30,31]. In a recent study, increased activity of circulating extracellular vesicles (microparticles) that promote TF expression was observed and was significantly correlated with plasma tPA and PAI-1 levels during HFRS. In particular, extracellular TF activity peaks in patients with DIC compared with those without DIC [32]. Most of the recently identified biomarkers that predict disease severity in PUUV infections are associated with variables reflecting thrombin formation and coagulation. As a result of hantavirus infection, coagulation is mostly abnormal, with complex status changes, and the coagulation system may be interconnected with the immune system through complement and inflammatory mediators, allowing considerable diversity and heterogeneity in the clinical course and prognosis, leading to differences in disease progression and outcomes in HFRS patients [29].

At present, it is still common in clinical practice to use symptomatic signs such as oliguria, anuria and renal injury to initially determine the severity of HFRS [33]. In conclusion, this study confirmed that it is possible to predict disease progression by monitoring coagulation indices and even to perform preliminary risk stratification of HFRS patients. Risk stratification at the early stage of HFRS can help clinicians identify critically ill patients at an early stage, make more appropriate therapeutic decisions, and optimize the matching of patients with medical assistance that is appropriate for their condition while reducing the waste of healthcare resources. In addition, several other defining factors of critical HFRS, such as death, shock, bleeding requiring transfusion, or admission to the intensive care unit, are not necessarily associated with AKI [34], and these disease manifestations clearly fall under critical HFRS as well. We analysed the risk of critical HFRS via retrospective analyses in the form of a composite of temperature, oliguria symptoms, hypotensive shock symptoms, degree of bleeding, and comorbidities with other organ failure, and other conditions were synthesized to determine the patient’s condition without the omission of other severe form. Moreover, the indicators that independently predicted critical outcome at the time of the first medical contact, K, ALB, CK, SCR, angle, WBC and LYM were included in the risk model of critical illness and further quantified to make the judgment of critical illness more operable. The model had a high discriminatory value (AUC of 0.9058) (Fig 2A), which was superior to the prediction of progression (to critical illness) by any single parameter (Table 4). The model had acceptable discriminatory power (C index, 0.866) and good calibration (GOF P = 0.825). The externally validated AUC was 0.762 (Fig 2E). The calibration curve results revealed that the model-predicted incidence of critical illnesses matched reasonably well with the observed incidence of critical illnesses in the study population (Fig 2B). Decision curve analyses graphically revealed significant clinical utility and comparable net benefits of the model at different threshold probabilities (Figs 2C **and** 2D). For optimal predictive value and clinical benefit, we recommend 17.30% as the threshold for predicting the risk of criticalization in patients with HFRS and transferring them to an intensive care unit or a higher-level hospital for more comprehensive and effective medical support when appropriate. In recent years, several studies have identified several early predictors associated with the severity and prognosis of HFRS, such as urinary neutrophil gelatinase-associated lipid carrier protein, pentraxin-3 (PTX3) and so on.[19,35]. PTX3 is a soluble pattern-recognition receptor that participates in innate host defences against certain pathogens, and high PTX3 levels are associated with severe thrombocytopenia, and there is a correlation between high levels of PTX3 and several variables responsive to coagulation (low levels of the AT, PS, and PC). By acting through cross-linking of coagulation and complement system activation, PTX-3 is thought to play a role in disease pathogenesis [36]. A review also summarized immunoinflammatory biomarkers of the severity of PUUV hantavirus infection [37]. For example, in PUUV-infected patients, high plasma levels of IL-6 were associated with more severe disease [38]; in acute PUUV infections, elevated levels of indoleamine 2,3-dioxygenase (IDO) were associated with more severe AKI, more pronounced inflammation, and longer hospitalization in acute PUUV infections [39]; and in HTNV-induced HFRS, plasma cf-DNA levels were associated with HTNV loads and disease severity [40]. However, almost all of these mentioned biomarkers require special laboratory platforms, are generally difficult to measure, or are costly, and testing for these parameters is usually not available in clinical practice. In our study, ALB, SCR, CK, K, Angle, WBC, NE, and LYM were readily available markers of poor outcomes and can be readily applied in primary health care settings.

However, this study also has limitations. First, this was a single-center, retrospective study, and the selection of participants was clearly limited by the format of this study, leading to selection bias, while a retrospective cohort carries a high risk of missing data. Second, the small sample size and lack of spatial external data validation were also limitations of our study, and the validity of the model still needs to be assessed by further clinical studies in other endemic areas. Therefore, the validity and stability of the model must be demonstrated in a representative large sample, and prospective and validation studies are required for statistical analysis.

In summary, with the gradual worsening of the disease in HFRS patients, the haemostatic balance of the body will exhibit a corresponding weakening of coagulation ability and bleeding tendency. Disease progression can be predicted by monitoring the coagulation index. In our study, we used laboratory parameters observed at the time of the first medical contact, corrected for multiplicity, and combined them with thromboelastography examination indices to propose a simple and practical model for the assessment of criticalization, which is useful for the early preliminary assessment of a patient with HFRS, guiding treatment and prognostic assessment.

## Supporting information

S1 Data Training cohort. (XLSX)

S2 Data Validation cohort. (XLSX)
